# Considerations in Preparative Regimen Selection to Minimize Rejection in Pediatric Hematopoietic Transplantation in Non-Malignant Diseases

**DOI:** 10.3389/fimmu.2020.567423

**Published:** 2020-10-19

**Authors:** Robert J. Hayashi

**Affiliations:** Division of Pediatric Hematology/Oncology, Washington University School of Medicine, St. Louis, MO, United States

**Keywords:** transplantation, preparative, childhood and adolescence, hematopoeific stem cells, engrafted survival outcomes

## Abstract

The variables that influence the selection of a preparative regimen for a pediatric hematopoietic stem cell transplant procedure encompasses many issues. When one considers this procedure for non-malignant diseases, components in a preparative regimen that were historically developed to reduce malignant tumor burden may be unnecessary. The primary goal of the procedure in this instance becomes engraftment with the establishment of normal hematopoiesis and a normal immune system. Overcoming rejection becomes the primary priority, but pursuit of this goal cannot neglect organ toxicity, or post-transplant morbidity such as graft-versus-host disease or life threatening infections. With the improvements in supportive care, newborn screening techniques for early disease detection, and the expansion of viable donor sources, we have reached a stage where hematopoietic stem cell transplantation can be considered for virtually any patient with a hematopoietic based disease. Advancing preparative regiments that minimize rejection and transplant related toxicity will thus dictate to what extent this medical technology is fully utilized. This mini-review will provide an overview of the origins of conditioning regimens for transplantation and how agents and techniques have evolved to make hematopoietic stem cell transplantation a viable option for children with non-malignant diseases of the hematopoietic system. We will summarize the current state of this facet of the transplant procedure and describe the considerations that come into play in selecting a particular preparative regimen. Decisions within this realm must tailor the treatment to the primary disease condition to ideally achieve an optimal outcome. Finally, we will project forward where advances are needed to overcome the persistent engraftment obstacles that currently limit the utilization of transplantation for haematopoietically based diseases in children.

## Introduction

Since its first attempts in the 1950s, allogeneic hematopoietic stem cell transplantation (HSCT) has rapidly evolved over time (1). Initially used for the most desperate of situations, it has now become a standard of care for many disease conditions. This transformation is a product of many advancements including: (1) Improving our understanding of hematopoiesis and immune reconstitution. (2) Improvements in supportive care, (3) Improvements in the prevention of graft-versus-host disease (GVHD), (4) Expansion of donor pools, (5) Refinements in preparative regimen selection and design. These advancements have produced a steady decline in transplant related mortality rates which now approach 10% in some instances. Thus, HSCT is now viewed as a viable option for virtually any disease that originates from the hematopoietic system. Continued improvements must now take into account not only mortality, but also minimizing the long-term toxicities that a surviving patient must confront after achieving cure of their primary disease.

Long term toxicities can be a consequence of several variables. 1.) Organ damage from the preparative regimen, 2.) Sequelae from the transplant course such as mucositis, infection, or excessive bleeding, 3.) Chronic GVHD, 4.) Toxicity from other medications administered (calcineurin inhibitors, steroids, etc.) (2). Although some of these complications may be unpredictable, the choice of the preparative regimen can have a significant impact. For non-malignant conditions, the primary goal of the transplant procedure is to achieve stable engraftment that is sufficient to rectify the underlying disease yet minimize long term toxicity (3). In its simplest view, the primary obstacle of HSCT is rejection of the graft. Thus, the choice of preparative regimen should focus on its immunosuppressive properties, optimizing engraftment yet avoiding an excessive immunocompromised state leading to life threatening infections (4). This “balance” can be difficult to achieve, and the optimal regimen, which varies with the primary disease, has not been established for any condition.

This mini-review will summarize both the history and current state of the repertoire of preparative regimens that have been utilized for HSCT for non-malignant conditions. We will discuss the variables which should be considered in choosing the appropriate preparative regimen and how different conditions may warrant different approaches. Finally, we will discuss future directions where advances in preparative regimen design may improve the outcome for these patients.

## Individual Agents Utilized for Preparative Regimen Design

Established preparative regimens have historically been developed utilizing standard phase I designs which advance dose intensity until a dose limiting toxicity was encountered. Hematologic toxicity was disregarded due to its reversal with the infusion of hematopoietic stem cells of the graft. Thus, doses and schedules of individual agents were limited by toxicities outside the hematopoietic system.

Modern day regimens are typically classified into three categories (3, 5, 6). *Myeloablative regimens* typically requires a stem cell graft infusion to reconstitute hematopoiesis. *Non-myeloablative regimens*, as the name implies, are less intensive and, even in the absence of a stem cell infusion, spontaneous hematopoietic recovery is expected. *Reduced intensity regimens*, whose definition has not been rigorously defined, falls somewhere in-between the two extremes, and is an acknowledgement that non-myeloablative regimens are associated, by their nature, with an increased risk of rejection. Reduced intensity regimens thus, fall short of full myeloablative dosing, but may achieve engraftment with less toxicity. Regardless of the type of preparative regimen, below are the components which constitute most modern day therapies.

### Total Body Irradiation (TBI)

One of the first modalities developed, TBI was the primary modality utilized in early transplant studies in animals because of its known immunosuppressive and myeloablative properties (7, 8). Clinical experience in humans quickly raised awareness of TBI’s effects on the lungs and strategies that fractionated doses and shielded the lung fields led to improvements in survival (9). TBI’s toxicity unfortunately does not spare any tissue, often leading to irreversible damage to exposed organs making it less attractive for non-malignant diseases. Subsequent investigations have strived to reduce the dose and presumably the toxicity to exposed organ systems because of its usefulness in overcoming rejection particularly in mismatched donors. Long term studies have failed to identify doses that are free of significant rates of infertility, thyroid disease, and growth hormone deficiency making the use of this modality problematic.

### Cyclophosphamide

A well-established alkylating agent, cyclophosphamide has maintained its role in HSCT due to its highly immunosuppressive properties and the relative resistance of hematopoietic stem cells to this agent even the highest doses (8, 10, 11). Recent studies have utilized cyclophosphamide post graft infusion to improve the outcomes of haploidentical transplant procedures (12–14). The success of this strategy has probably entrenched this agent as a major element of transplant therapy. Acute toxicities including hemorrhagic cystitis, and cardiac toxicity have been reduced with improved supportive care, with persistent long term toxicities that include sterility and secondary malignancies.

### Busulfan

One of the first agents to be utilized in non-TBI containing preparative regimens, the establishment of pharmacokinetic modeling to project optimal dosing for this drug has reduced rejections and hepatotoxicity (8, 10, 11, 15). Seizures, a common complication of this agent has been minimized with prophylactic anti-epileptic drugs. Sinusoidal obstruction syndrome, (SOS) continues to be a clinical problem, but pharmacokinetic dose adjustments have reduced its risk.

### Treosulfan

A structural analog of busulfan, its use is increasing with its potent immunosuppressive properties and favorable toxicity profile (16–19). Future trials will determine whether it supplants busulfan as a primary agent for preparative regimens.

### Thiotepa

An alkylating agent, thiotepa has gained increasing popularity due to its immunosuppressive effects and its ability to lower rejection rates in reduced intensity preparative regimens (8, 20, 21). Its toxicity profile is comparable to other alkylating agents although it does have unique properties that lead to significant cutaneous toxicity which is typically managed with supportive care.

### Melphalan

Another popular alkylating agent, its use has increased over the years as its toxicity is limited outside of the hematopoietic system particularly at doses used in modern reduced intensity regimens (22).

### Etoposide

A phase specific, topoisomerase II inhibitor, etoposide has continued to be a common component of modern day preparative regimens due to its predictable toxicity profile and its ability to be combined with alkylating agents without adding excessive side effects (8). Most short term toxicities outside of myelosuppression has been restricted to gastrointestinal and dermatologic which can be typically managed, and severe liver toxicity is observed only with high doses (23). Etoposide’s association with an increased risk of secondary leukemia limits its use and makes it a somewhat less attractive agent for transplantation in non-malignant conditions.

### Fludarabine

A purine analog, fludarabine’s popularity in its incorporation into more modern day preparative regimens is due to its relatively potent immunosuppressive properties without significant organ toxicity (10, 11, 24). Early use of this agent was associated with neurologic toxicity which has been overcome with dosing adjustments. Its successful incorporation into several reduced intensity preparative regimens for non-malignant diseases would indicate that it will a remain central element in HSCT for the foreseeable future.

### Antibody Agents

Antibodies directed at the lymphoid compartment have an inherent attractiveness due to their lack of toxicities on other organ systems (3). Such agents can help overcome rejection. In addition, their typical long half-life allows for its persistence in the recipient where it can potentially impact GVHD, depleting T cells from the infused donor product. Appropriate premedication can overcome most infusion reactions. The greatest challenge is to tailor the dosing and schedule of administration to minimize rejection yet avoid sustained suppression of the T cell compartment that would lead to excessive opportunistic infections. Although many agents have been utilized over the years, only a few have maintained a stable presence in this field.

#### Anti-Thymocyte Globulin (ATG)

Two sources of anti-thymocyte globulin encompass most of its use: 1) ATGAM (horse polysera) 2. Thymoglobulin (rabbit polysera). ATGAM has been utilized for many more years than the rabbit formulation (25), but the latter is a more potent agent (26, 27). Studies with ATGAM have demonstrated that its use reduces the duration of other immunosuppressive agents (28). Both have been shown to improve engraftment rates when added to conventional preparative regimens and given their retained presence in the host, their use has reduced rates of both acute and chronic GVHD to varying degrees (29–32).

#### Anti-T Lymphocytes Globulin (ATLG)

Anti-T lymphocytes globulin, derived from rabbit polysera from immunization with a Jurkat T cell leukemia line, is also gaining in popularity (27, 33, 34). Most trials comparing the efficacy between ATG and ATLG have been performed in patients with malignant disease where more effective lymphodepletion and subsequent reductions in GVHD have been offset by increased rates of relapse of the primary cancer (35). More robust trials in non-malignant diseases are needed.

#### Alemtuzumab

A humanized monoclonal antibody against CD52, alemtuzumab has been shown to target T and B cells, NK cells, and antigen-presenting cells. It has been incorporated into several reduced intensity preparative regimens and has been used successfully for immunodeficiencies, hemophagocytic lymphohistiocytosis, lysosomal storages disease, thalassemia and sickle cell disease. Like other anti-lymphocyte products, it is associated with an increased risk for infections (36). However, since it is a monoclonal product, the clinical responses may be less variable from patient to patient in comparison to the polyclonal products listed above.

#### Co-Stimulation Blockade

Recent investigations have begun to examine T cell co-stimulation blockade as an additional means of immunosuppression to both reduce the risk of rejection and GVHD. Abatacept, a CTLA4-Ig agents can block the CD28-CD80/86 interactions needed for T cell activation has been incorporated into newer preparative regimens (37). Preliminary studies have demonstrated low rates of GVHD with an acceptable toxicity profile. Further trials are needed to further define its role.

### Agents Less Commonly Used in Preparative Regimens for Non-Malignant Disease

Other chemotherapy agents which were initially advanced into preparative regimens have not sustained their presence in modern day treatments for non-malignant diseases due to their inherent toxicities and the lack of a need for their anti-neoplastic activity. Platinum agents, other alkylating agents, anthracyclines, are examples of agents that have not sustained their presence in modern day regimens (8).

### Strategies in Preparative Regimen Selection for Non-Malignant Diseases

Lacking the necessity of eradicating malignant cells, the transplant physician contemplating HSCT for a patient with a non-malignant disease must take several considerations into account which may or may not be specific to the patient’s disease state. These include: 1) What are the specific vulnerabilities of a particular disease population that lead to transplant related complications from the preparative regimen selection? 2) How has the patient’s primary disease and the corresponding treatment to treat that disease impacted the patient’s vital organs? 3) What are the barriers to achieve engraftment which would guide minimizing the intensity of the preparative regimen? 4.) What are other immunological features beyond rejection that influence transplant outcome? Thoughtful consideration for each of these variables will optimize the course of the patient.

#### Specific Vulnerabilities of a Particular Disease Population

The different diseases which are considered for HSCT have different clinical phenotypes which are linked to problems, some which are severe. Although a successful HSCT procedure may ultimately alleviate the condition, specific elements of a particular preparative regimen may exacerbate a patient’s clinical condition to serious levels. An appreciation of the specific vulnerabilities for a particular disease will provide insight for thoughtful decision making to select a preparative regimen (**Table 1**). Given the diversity of clinical difficulties that each disease possesses and given the expected patient to patient variability in clinical courses, having a transplant team with sufficient experience for a particular disease will ensure optimal management of the unique complications that a patient may experience.

**Table 1:** Disease-specific vulnerabilities and the influence of preparative regimens on HSCT course.

Disease	Specific vulnerabilities	Impact of preparative regimen toxicities	Agents to be used with caution	Agents with less associated toxicity
SCID (22, 38–42)	Pre-existing infection	Disruption of mucosal barriers Prolonged myelosuppression/ immunosuppression Pulmonary toxicity/Pneumonitis	TBI, High-dose busulfan,	Fludarabine Dose adjusted busulfan Cyclophosphamide Melphalan Lymphocyte depleting antibodies (ATG, ALG, Alemtuzimab)
Other immunodeficiencies (22, 43–49)	Pre-existing infection Autoimmune disease Higher rates of rejection	Disruption of mucosal barriers Prolonged Myelosuppression /immunosuppression Autoimmune cytopenias Pulmonary toxicity/Pneumonitis	TBI, High-dose busulfan,	Fludarabine Dose adjusted busulfan Treosulfan Cyclophosphamide Melphalan Lymphocyte depleting antibodies (ATG, ALG, Alemtuzimab)
Chronic granulomatous disease (21, 50–52)	Chronic aspergillus pneumonitis Granulomatous lung disease Inflammatory bowel disease Anti-Kell alloimmunization	Pulmonary toxicity/Pneumonitis Fungal sepsis. Bowel injury	TBI High-dose busulfan	Fludarabine Dose adjusted busulfan Treosulfan Cyclophosphamide Melphalan Lymphocyte depleting antibodies (ATG
Aplastic Anemia (53–56)	Blood product sensitization Iron overload Chronic neutropenia/infection	Mucositis SOS Hemorrhagic cystitis	TBI	Cyclophosphamide Lymphocyte depleting antibodies (ATG, ALG, Alemtuzimab)
Fanconi’s Anemia (57–63)	Poor DNA repair Endocrine deficiencies MDS/AML	Mucositis SOS Pulmonary toxicity/Pneumonitis Renal insufficiency Hemorrhagic cystitis	Radiation, Alkylating agents	Dose adjusted busulfan Cyclophosphamide Fludarabine ATG
Inherited Bone Marrow Failure Syndromes, other than Fanconi’s anemia (19, 64, 65)	DNA repair defects (DKC) Endocrinopathies Chronic neutropenia/infection	Severe mucosal injury Pulmonary toxicity SOS Infection Hemorrhage	TBI, high dose Alkylating agents	Fludarabine Cyclophosphamide Melphalan Lymphocyte depleting antibodies (ATG, ALG, Alemtuzimab)
Leukodystrophies (66–70)	Leukoencephalopathy, Adrenal insufficiency	Seizures, decline in neurologic and cognitive function, Adrenal insufficiency (ALD) Swallowing difficulties, Impaired ambulation	Radiation High dose busulfan	Dose adjusted busulfan Cyclophosphamide Fludarabine Lymphocyte depleting antibodies (ATG, ALG, Alemtuzimab)
Hurler’s Disease (66, 71–73)	Upper airway patency, Heart failure	Mucositis, Airway obstruction	Radiation	Dose adjusted busulfan Cyclophosphamide Fludarabine Lymphocyte depleting antibodies (ATG, ALG, Alemtuzimab)
Thalassemia (74–80)	Iron overload	Mucositis SOS Pulmonary toxicity/Pneumonitis Hemorrhage	Radiation High-dose busulfan	Dose adjusted busulfan Cyclophosphamide Fludarabine Treosulfan Lymphocyte depleting antibodies (ATG
Sickle cell anemia (81–85)	History of stroke/vasculopathy Recurrent Chest Syndrome/Pulmonary compromise Renal insufficiency Red cell alloimmunization	Mucositis Seizures PRES Renal injury	Radiation High-dose busulfan	Dose adjusted busulfan Cyclophosphamide Fludarabine Lymphocyte depleting antibodies (ATG, ALG, Alemtuzimab)

ATG, anti thymocyte globulin; ALG, anti-lymphocyte globulin; PRES, posterior reversible leukoencephalopathy syndrome; SOS, inusoidal obstruction syndrome; TBI, total body irradiation.

##### How Has the Patient’s Primary Disease and the Corresponding Treatment to Treat That Disease Impacted the Patient’s Vital Organs?

The natural history of a particular disease may lead to organ compromise that may make the patient less tolerant to preparative regimens with specific toxicities. For instance, patients with leukodystrophies with substantial demyelination of the CNS may not tolerate TBI or high doses of neurotoxic chemotherapy such as busulfan (66, 67). A patient with sickle cell disease who has acquired substantial renal injury may handle agents cleared by the kidney poorly leading to heightened toxicity (81, 82). Alternatively, a patient with an immune compromised state such as chronic granulomatous disease may have incomplete clearance of infections which may worsen and progress once the full immunosuppressive effects of the preparative regimen have taken hold (50, 51). Thus, not only must the clinician be sufficiently familiar with the inherent vulnerabilities of the patient’s disease state, but an evaluation that sufficiently characterizes an individual’s susceptibilities to the procedure is a critical facet of the process. Preparative regimen selection and agent dosing may need to be individualized for a patient to minimize the toxicities while still striving toward a successful procedure. A sensitivity to these issues will minimize the transplant related morbidity and mortality for the patient, who could otherwise survive for a substantial number of years in the absence of the transplant procedure.

##### What Are the Barriers to Achieve Engraftment Which Would Guide Minimizing the Intensity of the Preparative Regimen?

The barriers to engraftment are primarily immunologic, with its magnitude dictated by the patient’s underlying disease and past treatment history (54, 57, 71). Certainly immunodeficiencies are presumed to be less capable of rejecting infused grafts, but there is wide variability in the immune competence between primary diagnoses and even for patients with the same disease. This may not necessarily be reflective in obvious differences in phenotype, but it will manifest itself in rejection (43–45). There is a tendency to provide as minimal intensity as possible for patients with immunodeficiencies to try and reduce toxicities, particularly if the patient presents with a preexisting infection. However, rejections from an inadequate preparative regimen will invariably lead to a need to repeated procedures of increasing preparative regimen intensity to avoid another rejection. Such escalation will invariably result in the accumulation of toxicities potentially leading to an unsatisfactory result.

Other disease states that are amenable to HSCT may in fact have intact immune systems. In contrast to patients with malignancies in which prior chemotherapy exposure may reduce the likelihood for rejection, non-malignant diseases, such as lysosomal storage diseases, leukodytrophies, and hemoglobinopathies may require preparative regimens with substantial immunosuppressive properties, perhaps even requiring fully myeloablative regimens (20, 66, 71, 72, 82). Such transplant procedures will lead to more severe long term toxicities.

Conditions of bone marrow failure further illustrate the complexities of choosing the right preparative regimen. Aplastic anemia, typically a disease of T cell mediated destruction of the hematopoietic system, is a condition where prior blood product exposure may sensitize the donor to an even greater risk of rejection (55). Alternatively, other conditions such as Fanconi’s Anemia or Dyskeratosis Congenita, possess difficulties in DNA repair with intolerance to the even most modest doses of radiation or alkylating agents (57–60, 64). Thus, even conditions of poor marrow function present with a wide array of clinical challenges.

##### What Are Other Immunological Features Beyond Rejection That Influence Transplant Outcome?

Beyond rejection, the immune system plays a central role in the clinical course of the transplanted patient. The expansion of alloreactive T cells will ultimately result in varying degrees of GVHD, and will have a substantial impact on both long term toxicity and treatment related mortality. Simultaneously, the newly reconstituting immune system is striving to achieve a protective state against infections, building new B and T cell repertoires while priming to new antigens (38, 86–90). Further complicating this process is the impact specific preparative regimen agents may have on the newly emerging lymphocyte population. Antibodies with specificity to different lymphocyte populations (ATG, ATLG, alemtuzumab etc.) will linger in the body many days after their infusion and impact not only the infused lymphocyte populations of the graft but also the newly emerging populations. The amount of antibody present as the engrafting lymphocytes develop varies with the agent, dose administered, and between patients. Thus, the transplant physician must use information from past clinical trials in selecting the appropriate regimen for an individual patient in contrast to making empiric decisions. A reduced effect on the emerging immune system may lead to extensive GVHD, while an excessive one may lead to life threatening infections (91). The inability to “fine tune” this effect is a limiting feature of the use of antibody agents.

##### Thoughtful Use of Preparative Regimens in HSCT in Non-Malignant Diseases

It is apparent from this review that many challenges confront the clinician when choosing a preparative regimen for a transplant candidate. Over the past several decades, investigators have reported their successes and challenges exploring different strategies (**Table 2**). It is apparent that virtually every element of the transplant course from rejection risk to overall survival vary tremendously from report to report. Furthermore, variables such as donor source, age of the patient, and disease status prior to the transplant procedure can influence the transplant outcome further obscuring the impact of the preparative regimen. This variability is in part due to differences in the condition of the patient population transplanted, the agents used to formulate the preparative regimen, the graft selection, (matched sibling, matched or mismatched unrelated donor, cord blood, peripheral blood verses bone marrow), and graft manipulation (T cell depletion) which will result in varying outcomes. Furthermore, many reports merge outcomes of several different preparative regimens or combine multiple diseases together, sometimes making it impossible to link specific outcomes from a preparative regimen to a specific disease. Thus, comparisons between reports can be difficult. Programs and groups that commit to a specific preparative regimen “backbone,” and then refine elements from this backbone in well-defined cohorts will provide the most useful information on how to select a preparative regimen for a patient.

**Table 2:** Variation of HSCT outcomes.

Disease	Successful Preparative Regimens (#patients)	Graft Failure/Rejection Rate	aGVHD	cGVHD	TRM	EFS	OS
SCID (22, 38–42)	Range of Reported Outcomes^0,51,55-58^ None (21) (92) Bu/Cy (9) (93) Flu/Mel (5) (94) Bu/Cy/ATG (6) (95) Bu/Flu/ATG Treo/Flu Treo/Cy	0–82% 42%* 11% 0% 0%	0–65% 38% 22% 60% 50%	0–39% 0% 22% 33.3% -	0–24% 0% 33% 20% 33%	60–95% 95% 67% 80% 67%	67–84% 95% 67% 80% 67%
Other immunodeficiencies	Range of Reported Outcomes ^22,47,48,67-70^ Bu/Cy (7) (93) Alem/Treo/Flu (13) (48) Treo/Flu/Thio/RTX/ATG (8) (48) Alem/Flu/Mel (12) (46) Flu/Mel/ALG (5) (22) Bu/Cy/PTN Bu/Cy/ATG Bu/Flu/ATG Treo/Flu/	0–66.7% 0% 0% 12.5% 66.7% 20%	17.4–87.5% 57% 62% 87.5% - 50%	0–20% 14.2% 0% 0% - 20%	0–44% 14.2% 0% 12.5% 25% 20%	33–100% 86% 100% 87.5% 33% 80%	62.5–94% 86% 100% 87.5% 62.5% 80%
Chronic granulomatous disease (21, 50–52)	Range of Reported Outcomes^9,34,35,63^ Bu/Flu/ATG (96) Bu/Flu/Alem (96) Bu/Alem(5) (52) Bu/Alem/LD TBI (33) (52) Treo/Flu (5) (21) Treo/Flu/Alem (22) (21) Treo/Flu/Thio/ATG(5) (21) Alem/Flu/Mel (4) (97) Bu/Cy Bu/Cy/ATG Bu/Flu/Cy/ATG	0–20% 0% 9% 0% 12% 0% 13% 20% 25%	4–60% 33% 39%- - - 60% 40% 40% 50%	0–20% 4.8% 9% - - 20% 14% 0% 25%	0–40% 4.8% 6% 0% 3% 40% 4.5% 0% 0%	80–91% 97.2% 91% 100% 76% 60% 90% 80% 75%	60–100 97.2% 91% 100% 85% 60%% 95% 100% 75%
Aplastic Anemia (53–56)	Range of Reported Outcomes (7, 40, 41, 51) Cy/ATG (33) (98) Flu/Cy/ATG (28) (55) Flu/Cy/ATG^#^ (29) (55) Alem/Flu/Mel (17) (99) Bu/Cy Bu/Cy/ATG Cy Cy/TBI	0–6% 6% 3.6% 3.4% 0%	8–37.5% - 35.7% 37.5% 29%	6–37.5% 35.7% 37.5% 35%	5.7–32.1% 12% 32.1% 3.5% 12%	64.3–93.1% 81% 64.3% 93.1% 88%	67.9–96.6 89% 67.9% 96.6% 88%
Fanconi’s Anemia	Range of Reported Outcomes^46,52,54,55,74,76^ Cy (109) (62) Cy/TAI/ATG (35) (58) Bu/Flu/Cy/ATG (45) (100) Flu/Cy/ATG (44) (60)	0–11% 4% 5.7% 2.2% 0%	6.7–23% 11% 23% 6.7% 27%	4–36% 5% 12% 6.7% 4%	5.7–44% 12% 5.7% 17.8% 29.5%	70.5–94% 88% 89% 77.8% 70.5%	53.6–94% 88% 89% 80% 70.5%
Inherited Bone Marrow Failure Syndromes, other than Fanconi’s anemia	Range of Reported Outcomes ^19,53,77^ Bu/Cy/ATG (101) Treo/Flu/ATG (14) (19) Alem/Flu/Mel (6) (102) Alem/Flu/Mel (11) (103) Bu/Flu/Mel Mel/Flu/Cy Flu/Cy TBI/Mel/Cy	0–17% 10% 0 0% 9%	9–70% 70% 43% 33% 9%	10–31% 10% 14% 16% 27%	7–33% 20% 7% 33% 18%	62–93% 70% 93% 67% 82%	63.3–93% 80% 93% 67% 82%
Leukodystrophies	Range of Reported Outcomes ^38,39,78-80^ Alem/Flu/Mel (7) (104) Bu/Cy/ATG (12) (105) Bu/Cy/ATG (27) (106) Bu/Flu/Cy/ATG (4) (107) Bu/Cy Bu/Flu/ATG	0–12% 14.2% 9% 11.1% 0%	31–44% 71.4% 40% 40.7% 75%	10–25.9% 0% 10% 25.9% -	0–44% 14.2% 25% 25.9% 0%	48–100% 85.7% 66.7% 66.7% 100%	52–100% 85.7% 66.7%% 74.1% 100%
Hurler’s Disease (71–73)	Range of Reported Outcomes ^6,42,73^ Bu/Cy (8) (108) Bu/Cy/ATG (7) (109) Bu/Cy/ATG (20) (21) Bu/Flu/Mel/ATG (8) (110) Alem/Flu/Mel (7) (104)	0–37.4% 12.5% 0% 15% 0% 14.2%	12.2–16% 12.5% 28.6% 25% 25% 71.4%	0–14.8% 0% 0% 10% 0% -	0–45.8%- 12.5% 0% 15% 0% 14.2%	41.2–100% 75% 100% 85% 100% 85.7%	60.8–100% 87.5% 100% 85% 100% 85.7%
Thalassemia (74–80)	Range of Reported Outcomes ^4-80^ Bu/Cy/ATG(12) (76) Bu/Flu/Cy/ATG (48) (75) Thio/Treo/Flu/ATG (60) (77) Thio/Treo/Flu (28) (76) Bu/Flu/Thio (8) Bu/Flu/Thio/Abet (24) (111) Alem/Flu/Mel (9) (112) Alem/Flu//Thio/Mel (33) (79)	0–16.7% 16.7% 0% 9% 7.1% 0% 0% 0% 3%	14–75% 16.7% 8.3% 14% 14.3% 75% 16.7% - 33%	2–40% 16.7% 8.3 2% 10% 25% 25% - 21%	0–37.5% 0% 0% 7% 21.4% 37.5% 0% 0% 18%	62.5–100% 83% 100% 84% 71.4% 62.5% 100% 100% 64%	62.5–100% 100% 100% 93% 78.5% 62.5% 100 100% 82%
Sickle cell anemia	Range of Reported Outcomes ^40,41 89-91^ Bu/Cy (22) Bu/Cy/ATG (16) Bu/Flu/Cy/ATG (14) (113) Bu/Flu/ATG (22) (84) Alem/Flu/Mel (29) (83) Alem/TBI (30) (85) Flu//Thio/Mel/ATG (10) (114)	0–18% 18%% 0% 14% 4.5% 0% 0% 10%	0–33.3% 9% 13% 14% 18% 10% 0% 20%	0–62% 4.5% 0% 14% 27% 10% 0% 10%	0–28 9% 0% 0% 9% 10% 0% 10%	69–100% 73% 100% 100% 86% 80% 97% 80%	79–100% 91% 100 (115) % 100% 91% 90% 97% 90%

Range of Reported Outcomes summarized in the first line of each category. Number of patients specifically cited marked by () following listed preparative regimen. Preparative regimens used in respective disease where the specific preparative regimen could not be directly attributed to a specific disease specific population are left blank. Outcome measures missing or not extractable from the report are designated with a “-” symbol aGVHD, acute graft-versus-host disease; cGVHD, chronic graft versus host disease; TRM, transplant-related mortality; EFS, event-free survival; OS, overall survival; Alem, alemtuzumab; ATG, anti thymocyte globulin; Bu, busulfan; Cy, cyclophosphamide; Flu, fludarabine; Mel, melphalan; Treo, treosulfan; Thio, thiotepa; TAI, thoraco-abdominal irradiation; TBI, Total Body Irradiation; LD TBI, Low Dose TBI.

*Includes patients who lost B cell but retained T cell function.

^#^Reduced dose Cyclophosphamide, increased Fludarabine.

Considerations of the vulnerabilities of the primary disease, the clinical status of the individualized patient, the essential needs of overcoming rejection yet temporizing GVHD and life threatening infections must all be weighed in making the appropriate decision for the patient. Unfortunately, despite over three decades of experience, there is no “formula” that can be utilized to assemble a combination of agents that will give a predictable outcome fulfilling the needs of both the clinician and the patient. Large scale studies with detailed reports of outcomes and toxicities provide our only resource to guide the clinician to make thoughtful decisions for their patient. Further research with well-designed clinical trials with full characterization of outcomes are needed to enhance our understanding of this topic.

## Author Contributions

The author confirms being the sole contributor of this work and has approved it for publication.

## Conflict of Interest

The author declares that the research was conducted in the absence of any commercial or financial relationships that could be construed as a potential conflict of interest.
